# Dynamic Field Assessment of Hip Adductor Function Using a Smartphone-Based Copenhagen Test: Reliability and Concurrent Associations with Isometric Strength in Amateur Football Players

**DOI:** 10.3390/sports14040125

**Published:** 2026-03-24

**Authors:** Aaron Miralles-Iborra, Tomas Urban, Javier De Los Ríos-Calonge, Jose L. L. Elvira, Juan Del Coso, María Isabel Tomás-Rodríguez, Casto Juan-Recio, Víctor Moreno-Pérez

**Affiliations:** 1 Department of Sport Sciences, Sports Research Centre, Miguel Hernandez University of Elche, Avda. de la Universidad s/n., 03202 Elche, Alicante, Spain; 2 Sport Sciences Research Centre, Rey Juan Carlos University, 28032 Fuenlabrada, Spain; juan.delcoso@urjc.es; 3 Institute of Health and Sport Sciences, Faculty of Health Sciences, Universidad Francisco de Vitoria, 28223 Madrid, Spain; 4 Translational Research Centre of Physiotherapy, Department of Pathology and Surgery, Faculty of Medicine, Miguel Hernandez University, 03550 Sant Joan d’Alacant, Alicante, Spain; mitomas@umh.es

**Keywords:** groin injury, hip adduction torque, wearable sensors, field-based testing, consistency, rate of force development

## Abstract

Assessing hip adductor muscle strength is important for identifying weakness or side-to-side imbalances associated with groin injury risk. Although the Copenhagen adductor exercise is widely used to evaluate adductor function, the quantification of strength-related outcomes using inertial sensors integrated in smartphones during this task has not been systematically examined. This study aimed to evaluate the reliability of a smartphone-based Copenhagen adductor field test and its associations with established isometric hip adductor strength assessments. Twenty amateur male football players (21.1 ± 3.2 years) completed two laboratory sessions separated by one week. The reliability of the smartphone-based Copenhagen test was assessed for endurance-related outcome (repetition count) and strength-related outcomes (mean repetition time and peak velocity) using intraclass correlation coefficients (ICC), standard error of measurement (SEM), and minimum detectable change (MDC). Participants also performed unilateral and bilateral isometric hip adductor tests using load cells to obtain isometric peak force (IPF) and rate of force development at 150 ms (RFD150). Associations were examined using Pearson correlation coefficients. The smartphone-based Copenhagen test showed ICC point estimates ranging from 0.63 to 0.83, although several 95% confidence intervals were relatively wide (ICC = 0.63–0.83; SEM = 6.7–18.5%). Endurance-related outcomes were not significantly associated with IPF or RFD150. In contrast, peak velocity showed low-to-moderate correlations with RFD150 (*r* = 0.48–0.63) and moderate correlations with IPF (*r* = 0.50–0.64; *p* < 0.05). These findings suggest that the peak velocity obtained during the Copenhagen adductor test may provide a practical field-based complement to conventional isometric assessments. However, given the moderate strength of the observed associations and the measurement error of peak velocity, these outcomes should be interpreted with caution and warrant further investigation.

## 1. Introduction

Hip adductor strength assessment has become a central focus in sports medicine due to its role in groin injury risk and rehabilitation in field-based team sports [1,2,3,4]. High-intensity actions such as rapid accelerations, decelerations, changes in direction, and kicking expose the groin region to substantial mechanical stress, particularly affecting the adductor longus muscle [5,6]. Reduced adductor strength has consistently been identified as a risk factor for groin-related problems and is therefore widely monitored in both clinical and performance settings [1,2,3,4,7].

Several devices and protocols have been used to measure the hip adductor muscle strength. Although isokinetic dynamometry is considered the gold standard, its limited portability and high cost restrict field application [8]. Consequently, handheld dynamometers [9], sphygmomanometers [10], and load cell-based systems [11,12] are commonly adopted in practice and demonstrate acceptable validity and reliability [13]. However, these tools primarily assess static force production and may be limited by inter-rater variability or practical constraints in applied environments.

The Copenhagen adductor exercise [14,15] has emerged as a widely implemented strengthening strategy and has recently been explored as an assessment tool for eccentric adductor capacity [12,16]. Given its functional and sport-specific characteristics, extending its application toward objective field-based monitoring may be clinically valuable. In parallel, inertial measurement units (IMUs) have gained attention for quantifying movement velocity and repetition characteristics during bodyweight exercises [17,18]. Despite this potential, no studies have examined whether velocity- or repetition-based metrics derived from IMUs during a Copenhagen adductor task align with established isometric hip adduction strength measures.

Therefore, the primary aim of this study was to evaluate the reliability of a smartphone-based Copenhagen adductor field test and to examine its associations with conventional isometric hip adductor strength assessments. Based on prior research [18], we hypothesised that velocity-based outcomes derived from the smartphone-based Copenhagen test would demonstrate acceptable reliability and show stronger associations with isometric peak force and rate of force development than endurance-related outcomes.

## 2. Methods

### 2.1. Participants

Twenty amateur male football players (mean ± SD: age 21 ± 3 years; body mass 70 ± 10 kg; height 1.75 ± 0.08 m) volunteered to participate. All participants were physically active and engaged in 1–3 h of combined football and resistance training, three to four times per week. Two participants did not complete the second testing session due to scheduling conflicts, resulting in incomplete datasets; therefore, the final analysed sample consisted of 18 participants. An a priori sample size calculation was performed using G*Power (version 3.1, Düsseldorf, Germany). Based on previous methodological recommendations and the comparable literature [19,20], a moderate expected correlation (*r* = 0.60) was assumed. Under these conditions, a minimum of 19 participants was required to achieve 80% statistical power at an alpha level of 0.05.

Inclusion criteria required participants to be aged between 18 and 35 years and free from lower-limb joint pathology (past six months), lower-limb muscle injury (past three months), lower-limb surgery (past 12 months), and any pain or discomfort at the time of testing. All participants were fully informed about the procedures and provided written informed consent prior to participation. The study was conducted in accordance with the Declaration of Helsinki and approved by the University Office for Research Ethics (code: DCD.JLE.01.20).

### 2.2. Procedures

Participants completed two testing sessions (approximately 30 min each) in a university-based biomechanics laboratory, separated by one week to minimise residual fatigue. Prior to testing, personal and medical history were recorded, along with anthropometric measurements including body mass, height, and lower-limb length (measured from the anterior superior iliac spine to the most prominent aspect of the medial malleolus) [21] (Table 1). Leg dominance was defined retrospectively as the limb producing the higher normalised isometric peak force during the unilateral hip adduction test. This approach was selected instead of self-reported kicking preference, as strength dominance was considered more relevant to hip adduction performance and injury screening, and may not necessarily coincide with functional preference in football players. However, this operational definition may introduce a degree of dependence in side-specific analyses and should therefore be interpreted with caution. Limb testing order was counterbalanced according to the kicking preference leg to minimise potential fatigue or order effects.

Before each session, participants performed a standardised football-specific warm-up consisting of dynamic running drills (two sets of 30 m each), including straight running, hip-out, hip-in, circling partner, shoulder contact, and rapid forward–backward movements, following established protocols [22]. All exercises were completed at a self-selected moderate intensity under investigator supervision to ensure consistency. Participants were familiarised with all procedures prior to data collection.

During the first session, participants performed three maximal repetitions of the unilateral isometric hip adduction test per limb (60 s rest between repetitions and limbs), followed by two 20 s sets of the Copenhagen adductor exercise per limb (2 min rest between sets). The starting limb was counterbalanced. The testing order was fixed, as the dynamic Copenhagen task was expected to induce greater fatigue than the isometric assessments.

In the second session, participants completed the supine squeeze test, followed by a repeated 20 s Copenhagen adductor task under identical conditions to assess test–retest reliability. For the subsequent analyses, the Copenhagen adductor variables obtained in the second session were used. These values were analysed in relation to the unilateral isometric hip adduction test obtained in the first session and the supine squeeze test obtained in the second session. Consequently, associations involving the unilateral isometric test represent between-session relationships, whereas associations with the supine squeeze test represent same-session comparisons. All sessions were conducted at the same time of day to minimise diurnal variation. Participants were instructed to refrain from caffeine intake and strenuous physical activity for 24 h prior to testing.

### 2.3. Measurements

#### 2.3.1. Smartphone-Based Copenhagen Adductor Field Test

**Figure 1:**
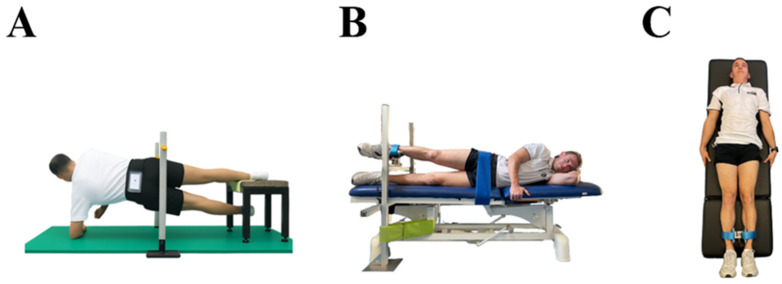
Assessment of hip adductor muscle strength tests: Copenhagen Adductor Exercise (**A**), unilateral hip adductor test (**B**), and supine squeeze test (**C**), using an inertial sensor (**A**) and a load cell (**B**,**C**).

The smartphone-based Copenhagen adductor field test was performed with participants in a side-lying position, supported on the forearm with the elbow vertically aligned under the shoulder. The test leg was placed on a 45 cm bench, while the contralateral leg remained extended and relaxed beneath it (Figure 1A). A vertical reference elastic band positioned at pelvic height provided external feedback. Participants were instructed to raise their hips until gently contacting the bar, ensuring consistent end-range positioning across repetitions. The standardised verbal instruction was: “Raise your hips as fast as possible, repeating the movement until exhaustion or until the tester’s signal after 20 s.” Participants were instructed to return fully to the starting position on each repetition, consistent with recommendations for dynamic endurance tasks [23]. The non-tested leg was maintained in a horizontal position to prevent momentum from limb swing. A smartphone (iPhone^®^ 7, Apple Inc., Cupertino, CA, USA) was secured at the lower lumbar region using an adjustable Velcro^®^ belt. This location was selected due to its proximity to the body’s centre of mass and reduced soft-tissue artefacts. Acceleration data were sampled at 200 Hz using the ForceData application (version 2.1.2, My Jump Lab, Madrid, Spain).

#### 2.3.2. Unilateral Isometric Adductor Strength Test

For the unilateral hip adductor strength test, participants were positioned in a side-lying position on a custom-built frame, with the test limb extended and placed above a load cell mounted on an adjustable-height bench (Figure 1B). The load cell was secured 10 cm above the medial malleolus of the test limb, while the contralateral limb remained extended and relaxed beneath the bench. The pelvis was stabilised using a strap to minimise compensatory movements [24]. A portable S-Type stainless steel load cell (Model 620 Tedea-Huntleigh, Vishay Precision Group Inc., Holon, Israel) was interfaced with Chronojump software (version 2.2.0-12, Chronojump Bosco System, Barcelona, Spain). The load cell was calibrated before each session using a standard 10 kg weight in accordance with manufacturer guidelines. Each trial consisted of a 5 s maximal voluntary isometric contraction following the verbal cue: “Contract as hard and as fast as possible and maintain the effort until instructed to relax.” Three trials were performed per limb with 15 s of rest between attempts [25].

#### 2.3.3. Supine Squeeze Test

For the supine squeeze test, participants lay in a supine position with both lower limbs extended. The load cell was positioned 10 cm proximal to the medial malleoli following established protocols [26] (Figure 1C). This long-lever configuration has been shown to effectively assess adductor longus function, and supine testing with fixed dynamometry demonstrates high reliability (0.87 ≤ ICC ≤ 0.96; SEM ≤ 14%) [26,27,28]. The test required a bilateral maximal adduction effort; therefore, unilateral values were not obtained. The same load cell and data acquisition system were used as in the unilateral protocol. Participants were instructed to avoid trunk or cervical flexion and rotation but were permitted to stabilise themselves by lightly gripping the stretcher.

### 2.4. Data Processing

Acceleration signals obtained from the smartphone’s built-in inertial sensor were processed using Python 3.7 through a custom pipeline. Raw tri-axial acceleration data were visually inspected and filtered using a fourth-order low-pass Butterworth filter with a cutoff frequency of 10 Hz, applied to signals sampled at 200 Hz, to attenuate high-frequency noise associated with soft-tissue artefacts. The resultant acceleration magnitude was calculated from the three orthogonal axes to reduce potential effects of device orientation. The filtered acceleration signal was subsequently integrated over time using a cumulative trapezoidal numerical integration to derive velocity profiles. To minimise potential integration drift, the analysis focused on relative peak values within each repetition rather than absolute velocity trajectories, and the signals were pre-filtered prior to integration.

Individual repetitions were automatically segmented based on velocity peaks using the Detecta event-detection library (version 0.0.5) [29], together with pandas (version 1.5.3) [30] and xarray (version 2022.12.0) [31]. Peak detection was performed using a minimum peak height threshold of 2 m·s^−2^ and a minimum peak distance of 60 samples (≈0.30 s at 200 Hz) to prevent multiple detections within the same repetition. Peak detection thresholds and minimum inter-peak distances were established through pilot testing to ensure accurate identification of concentric phases while minimising false detections. All signal processing and outcome extraction procedures were applied consistently across sessions and participants to ensure methodological reproducibility. These analysis windows were selected to capture the initial phase of the movement, where neuromuscular performance is least influenced by fatigue-related changes in movement execution. Furthermore, inertial outcomes were normalised to body mass and height to account for inter-individual differences in inertial demands and limb lever length during the movement.

The following inertial sensor-derived outcomes were calculated:

- Normalised repetition count (per height and body mass) during the first 10 s (m·kg^−1^): Repetition count was defined as the number of velocity peaks detected within the initial 10 s of the task. The total number of repetitions was normalised to participant height and body mass. Mean values across trials were retained for analysis.
- Mean repetition time (s): The average repetition duration was calculated from the first 10 repetitions by determining the time interval between consecutive velocity peaks. Mean values were retained for analysis.
- Normalised peak velocity (per height and body mass) during the first five repetitions (m·s^−1^·kg^−1^): Peak velocity was defined as the maximum value of the integrated velocity signal for each repetition. The mean of the first five repetitions was calculated and normalised to participant height and body mass. Mean values across trials were retained for analysis.

Isometric peak force (IPF) and rate of force development at 150 ms (RFD_150_) obtained from the load cell were automatically extracted using Chronojump software (version 2.2.0-12, Chronojump Bosco System^®^, Barcelona, Spain) based on fitted force–time curves for dominant and non-dominant limbs. RFD_150_ was calculated as the maximal slope of the force–time curve within the first 150 ms of contraction [32].

For subsequent analyses, the mean of three trials was retained for the unilateral hip adductor test and the mean of three maximal attempts was retained for the supine squeeze test. Values were normalised by dividing by body mass and multiplying by leg length to allow inter-participant comparison.

### 2.5. Statistical Analysis

All data were screened for normality using the Shapiro–Wilk test. Potential outliers were identified as values exceeding ±3 standard deviations from the mean. Descriptive statistics are presented as mean ± standard deviation, and statistical significance was set at *p* < 0.05. Absolute and relative between-session reliability of the inertial sensor-based Copenhagen adductor test outcomes were assessed using the standard error of measurement (SEM), relative SEM (SEM%), minimum detectable change (MDC_80_), and the intraclass correlation coefficient (ICC). SEM was calculated as the standard deviation of the paired differences divided by √2 and expressed relative to the mean outcome (SEM%). MDC_80_ was calculated as SEM × 1.28 × √2 (≈1.81 × SEM), providing a more realistic threshold for detecting real changes in applied sport contexts [33]. ICCs and their 95% confidence intervals (CIs) were estimated using a two-way mixed-effects model for absolute agreement (ICC [1,3]) as reliability was evaluated under a fixed testing configuration involving the same protocol, device setup, and analysis workflow across sessions. ICC values were interpreted using commonly cited descriptive thresholds; however, these categories were considered alongside the corresponding 95% confidence intervals, and outcomes with confidence intervals spanning multiple categories were interpreted cautiously. Reliability was interpreted as poor (<0.50), moderate (0.50–0.75), good (0.75–0.90), or excellent (>0.90) [34]. In addition, paired mean differences with 95% confidence intervals were calculated for the smartphone-based Copenhagen outcomes to evaluate potential systematic changes between sessions. Reliability metrics were computed using a validated spreadsheet tool (www.sportsci.org).

Three inertial sensor-derived outcomes were predefined a priori (repetition count, mean repetition time, and peak velocity). A minimum reliability threshold (ICC > 0.50) was applied as a quality-control criterion to support the interpretability of subsequent correlational analyses. All predefined outcomes met this threshold and were therefore retained. Statistical analyses were performed using custom scripts in Python 3.7 with the Pandas (v2.2.2), NumPy (v2.0.2), and SciPy (v1.15.3) libraries [30,35,36].

Pearson correlation coefficients (*r*) with 95% CI were calculated to examine associations between inertial sensor outcomes and load-cell-derived unilateral isometric measures (IPF and RFD_150_). Correlations were also calculated between equivalent outcomes across the unilateral and supine squeeze tests to assess inter-test alignment. Correlation magnitudes were interpreted as low (0.30–0.49), moderate (0.50–0.69), high (0.70–0.89), and very high (≥0.90) [37].

## 3. Results

Table 1 presents participant characteristics and normalised strength outcomes for the unilateral hip adductor and supine squeeze tests (mean ± SD).

Reliability analyses for the smartphone-based Copenhagen adductor field test showed ICC point estimates ranging from 0.62 to 0.83, although several 95% confidence intervals were relatively wide. For the endurance-related outcome, SEM ranged from 16.3% (dominant limb) to 17.7% (non-dominant limb), with ICC values of 0.76 and 0.62, respectively. Strength-related outcomes showed ICC values ranging from 0.63 to 0.83 and SEM values between 6.7% and 18.5% (Table 2). Paired mean differences (Session 2 − Session 1) showed a consistent directional change between sessions, with higher repetition count and peak velocity and lower mean repetition time in Session 2. The clearest between-session change was observed for mean repetition time, which was significantly lower in Session 2 for both limbs, whereas changes in repetition count and peak velocity did not reach statistical significance.

Given the established reliability, correlational analyses were conducted using data from the second assessment session. Table 3 presents associations between inertial sensor-derived outcomes and load-cell-derived unilateral isometric measures (IPF and RFD_150_).

No statistically significant relationships were observed between the endurance-related outcome and either IPF or RFD_150_ (*p* > 0.05). In contrast, peak velocity during the first five repetitions demonstrated moderate and statistically significant positive correlations with both IPF (dominant: *r* = 0.64; non-dominant: *r* = 0.50; *p* < 0.05) and RFD_150_ (dominant: *r* = 0.63; non-dominant: *r* = 0.48; *p* < 0.05). Although a small inverse trend was observed between mean repetition time and RFD_150_, these associations did not reach statistical significance (dominant: *r* = −0.43; non-dominant: *r* = −0.32; *p* > 0.05).

**Figure 2:**
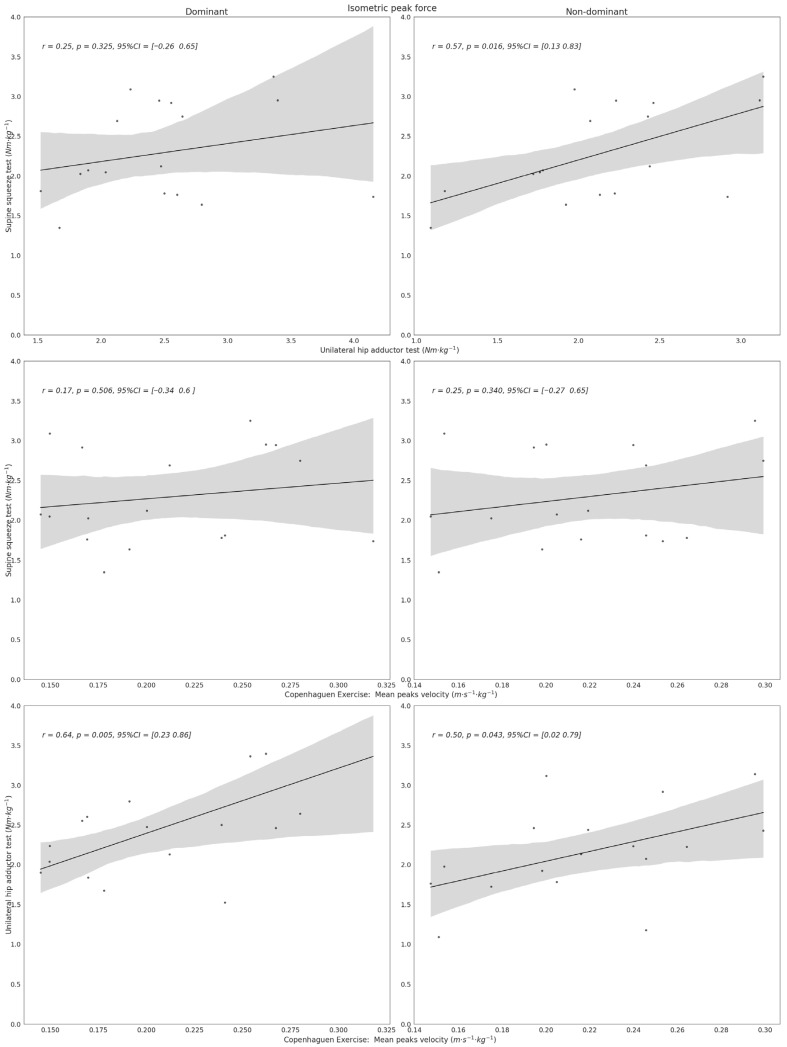
Alignment between isometric tests and Copenhagen adductor field-test outcomes with respect to isometric peak force (IPF). The figure displays the degree of association observed across test modalities. Dots represent individual observations, the black line indicates the linear regression fit, and the grey shaded area represents the 95% confidence interval.

Figure 2 illustrates the relationship between normalised peak velocity and IPF across tests. A moderate association was observed between the supine squeeze test and the non-dominant limb of the unilateral test (*r* = 0.57, *p* = 0.016). No significant association was found for the dominant limb, and no additional significant IPF associations were identified.

**Figure 3:**
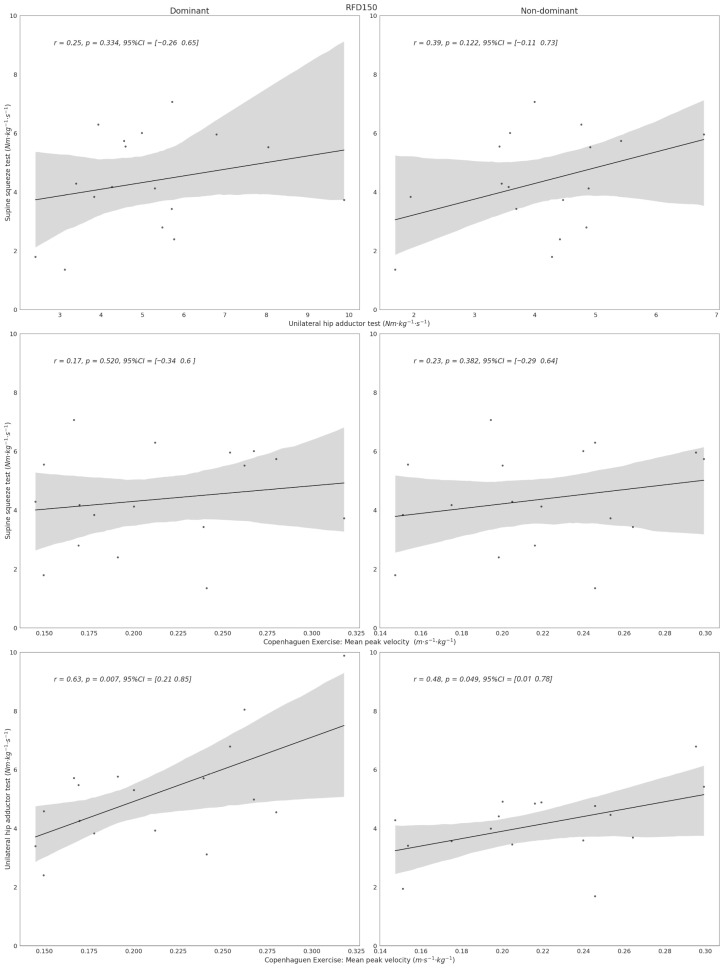
Alignment between isometric tests and Copenhagen adductor field-test outcomes with respect to rate of force development during the first 150 ms (RFD_150_). The figure displays the degree of association observed across test modalities. Dots represent individual observations, the black line indicates the linear regression fit, and the grey shaded area represents the 95% confidence interval.

Figure 3 presents equivalent analyses for RFD_150_. A low, non-significant association was observed between the supine squeeze test and the non-dominant limb (*r* = 0.39, *p* = 0.12), with no other significant inter-test relationships identified.

## 4. Discussion

The present study evaluated the reliability of a smartphone-based Copenhagen adductor field test and its association with established isometric hip adductor strength assessments. The main findings were: (1) ICC point estimates for strength-related outcomes ranged from 0.63 to 0.83, although several confidence intervals were relatively wide; (2) moderate associations between peak velocity and both IPF and RFD150, whereas endurance-related outcomes showed no significant relationships; and (3) limited agreement between dynamic and isometric tests, with greater agreement observed for IPF than for RFD_150_. These findings suggest that velocity-based metrics derived from the Copenhagen task capture related, but distinct, aspects of hip adductor function compared with traditional isometric assessments.

Although several field-based tools are available to assess hip adductor strength, most focus on maximal isometric force and require external fixation systems or examiner stabilisation [12,13]. The smartphone-based Copenhagen adductor field test does not aim to replace these instruments but to complement them by providing examiner-independent metrics within a functional movement pattern. In particular, quantifying execution velocity during repeated dynamic contractions may offer insight into rapid force expression and neuromuscular control, qualities not captured by static assessments. Given the eccentric and dynamic demands of football actions, peak velocity may reflect functionally relevant adaptations during training or rehabilitation. While the present study does not demonstrate superiority over existing tools, the minimal equipment requirements and feasibility of repeated field-based assessments support its applied relevance.

### 4.1. Reliability of the Smartphone-Based Copenhagen Adductor Field Test

Strength-related outcomes showed ICC point estimates ranging from 0.63 to 0.83; however, several confidence intervals were relatively wide and crossed multiple interpretative thresholds, indicating uncertainty around the exact level of reliability. Although these values (ICC = 0.63–0.83; SEM% = 6.7–18.5%) fall within ranges reported for other field-based strength assessments [12,13], the higher SEM observed for peak velocity indicates that small performance changes should be interpreted cautiously. From a practical standpoint, SEM and MDC_80_ provide useful benchmarks for interpreting repeated assessments, as small week-to-week fluctuations may reflect measurement variability rather than true performance changes, particularly for variables with greater relative error. Accordingly, practitioners should place greater confidence in changes that exceed the corresponding MDC_80_ threshold. This is especially relevant for peak velocity, for which measurement error was greater than for mean repetition time, suggesting that only changes of at least moderate magnitude are likely to reflect true performance changes rather than measurement variability [38]. In contrast, repetition count showed lower reliability, likely reflecting the greater influence of fatigue and pacing variability during endurance-based tasks.

### 4.2. Associations Between the Smartphone-Based Copenhagen Test and Unilateral Isometric Hip Adductor Test Measures

Moderate correlations between peak velocity and isometric strength measures suggest that both tests assess related, yet distinct, neuromuscular qualities, indicating partial overlap rather than equivalence between these assessments of hip adductor function. However, these tests likely reflected different physiological and mechanical demands. The smartphone-based Copenhagen test is a repeated dynamic bodyweight task that may depend on a combination of rapid force expression, movement coordination, trunk-pelvic control, and fatigue tolerance, whereas isometric strength tests assess maximal and early force production under fixed conditions. Therefore, peak velocity should not be interpreted as a direct surrogate of maximal isometric strength, and the smartphone-based Copenhagen test should be considered a complementary field-based assessment rather than a replacement for conventional isometric strength testing. These findings align with previous observations showing limited interchangeability between different hip adductor strength tests due to variations in body position, contraction type, and muscle recruitment patterns [13,39].

### 4.3. Alignment Between Different Isometric Strength Tests

Low-to-moderate alignment among isometric tests further supports the notion that different assessment protocols capture distinct mechanical and neuromuscular characteristics. Variations in lever length, contraction mode, body positioning, and unilateral versus bilateral execution likely explain the limited interchangeability observed between tests [40]. These findings are consistent with previous research reporting discrepancies between hip adductor assessment protocols despite targeting the same muscle group [13,39].

Notably, significant relationships between protocols were primarily identified in the non-dominant limb. This pattern may reflect bilateral deficit or bilateral facilitation phenomena, whereby force production differs when muscles contract simultaneously versus independently [41]. These mechanisms are thought to arise from neural and biomechanical factors influencing motor unit recruitment, interlimb coordination, and stabilisation demands [42]. Consequently, unilateral and bilateral tests may not activate the adductor musculature in an equivalent manner, which may partly explain the observed differences in alignment.

### 4.4. Strengths and Limitations

This study has several limitations that should be acknowledged. First, its cross-sectional design and the inclusion of non-injured amateur male football players preclude conclusions regarding injury risk prediction or return-to-play decision-making. Although the sample size was supported by an a priori power analysis, the findings should be interpreted within the context of a relatively small and specific sample of non-injured amateur male football players. As a result, the study was better suited to detect large associations than small-to-moderate ones, and the precision of some estimates was limited. Therefore, caution is warranted when interpreting weak or non-significant correlations and when generalising these findings to elite athletes, female players, or injured populations, in whom training background, neuromuscular characteristics, and tissue tolerance may differ substantially. A further methodological consideration is that limb dominance was defined retrospectively according to the limb showing greater normalised isometric peak force. Although this approach was intended to reflect functional strength dominance, it may have introduced partial statistical coupling in side-specific comparisons involving the same variable. Paired comparisons showed a consistent directional shift between sessions, with the clearest effect observed for mean repetition time, which was significantly lower in Session 2 for both limbs. These findings suggest that a residual familiarisation effect between sessions cannot be completely excluded despite the pre-test familiarisation. Finally, electromyographic data were not collected, limiting insight into muscle activation patterns. Future research should examine injured populations, female athletes, and elite players, and explore longitudinal relationships between dynamic and isometric strength adaptations.

### 4.5. Practical Applications

From a practical perspective, peak velocity derived from the smartphone-based Copenhagen test may provide a field-based indicator of dynamic hip adductor performance that shows partial association with conventional isometric strength measures. Given the moderate agreement with isometric assessments and the observed measurement variability, this tool should complement rather than replace established strength tests. As dynamic and isometric protocols appear to reflect distinct neuromuscular characteristics, practitioners should avoid directly comparing values obtained from different testing modalities and instead apply each assessment according to its specific purpose. The smartphone-based approach may be particularly useful for monitoring within-athlete changes across training or rehabilitation phases, especially when laboratory-based equipment is unavailable. Combining dynamic and isometric assessments may provide a more comprehensive profile of hip adductor function.

## 5. Conclusions

In summary, the smartphone-based Copenhagen adductor field test demonstrated moderate-to-good reliability for strength-related outcomes and showed ICC point estimates consistent with moderate-to-good reliability for strength-related outcomes, although several confidence intervals were relatively wide. Although these assessments are not interchangeable, wearable inertial systems may offer a practical and accessible complement for field-based monitoring. Further longitudinal research incorporating injury and performance outcomes is required before conclusions can be drawn regarding its predictive or preventive value.

## Figures and Tables

**Table 1:** Participants’ characteristics data.

		Mean	±	SD	Units
Age		21.06	±	3.24	years
Body mass		69.79	±	9.63	kg
Height		1.75	±	0.08	m
Leg length		0.90	±	0.05	m
Unilateral hip adductor test	Normalised IPF of non-dominant leg	2.2	±	0.6	N·m·kg ^−1^
	Normalised IPF of dominant leg	2.5	±	0.6	N·m·kg ^−1^
	Normalised RFD_150_ of non-dominant leg	4.3	±	1.2	N·m·kg ^−1^·s^−1^
	Normalised RFD_150_ of dominant leg	4.8	±	2.9	N·m·kg ^−1^·s^−1^
Supine Squeeze test	Normalised IPF	2.3	±	0.6	N·m·kg ^−1^
	Normalised RFD_150_	4.5	±	1.7	N·m·kg ^−1^·s^−1^

SD: standard deviation; IPF: isometric peak force; RFD_150_: rate of force development at 150 ms.

**Table 2:** Descriptive statistics and absolute and relative between-session reliability for the outcomes from the smartphone-based Copenhagen adductor field test.

			*n*	Session 1 (Mean ± SD)	Session 2 (Mean ± SD)	Change in Mean [95% CI]	SEM		MDC_80_ (%)	ICC Mean [95% CI]
							Mean [95% CI]	%		
Endurance-related outcome	Normalised repetition count (per height and body mass) during the first 10 s (m·kg^−1^)	D	18	0.51 ± 0.18	0.57 ± 0.14	0.055 [−0.004, 0.113]	0.08 [0.07, 0.12]	16.3	29.5	0.76 [0.53, 0.89]
		ND	18	0.49 ± 0.15	0.54 ± 0.12	0.051 [−0.010, 0.113]	0.09 [0.07, 0.12]	17.7	32.0	0.62 [0.30, 0.81]
Strength-related outcomes	Mean repetition time (s)	D	18	0.55 ± 0.11	0.47 ± 0.06	−0.080 * [−0.119, −0.040]	0.06 [0.04, 0.08]	10.3	18.6	0.65 [0.35, 0.83]
		ND	18	0.52 ± 0.09	0.47 ± 0.07	−0.046 * [−0.070, −0.022]	0.03 [0.03, 0.05]	6.7	12.1	0.83 [0.66, 0.92]
	Normalised peak velocity (per height and body mass) during the first five repetitions (m·s^−1^·kg^−1^)	D	18	0.21 ± 0.08	0.23 ± 0.07	0.024 [−0.003, 0.051]	0.04 [0.03, 0.05]	18.5	33.5	0.75 [0.51, 0.88]
		ND	18	0.20 ± 0.05	0.22 ± 0.05	0.022 [−0.0003, 0.045]	0.03 [0.03, 0.05]	16.0	29.0	0.63 [0.31, 0.82]

D: dominant leg; ND: non-dominant leg; *n*: number of subjects; SD: standard deviation; SEM: standard error of measurement; MDC: minimal detectable change; ICC: intra-class correlation coefficient; CI: confidence interval; * statistically significant *p* < 0.05.

**Table 3:** Relationships between outcomes from the smartphone-based Copenhagen adductor field test and the load cell-based unilateral isometric hip adductor strength test.

Smartphone-Based Copenhagen Test	Load Cell-Based Unilateral Isometric Hip Adductor Strength Test	Side	*r*	95% CI
Normalised repetition count (per height and body mass) during the first 10 s (m·kg^−1^)	Normalised isometric peak force (per leg length and body mass) (N·m·kg ^−1^)	D	0.18	[−0.31, 0.60]
		ND	0.13	[−0.35, 0.57]
Mean repetition time (s)		D	−0.28	[−0.66, 0.21]
		ND	−0.26	[−0.65, 0.24]
Normalised peak velocity (per height and body mass) during the first five repetitions (m·s^−1^·kg^−1^)		D	0.64 *	[0.23, 0.86]
		ND	0.50 *	[0.02, 0.79]
Normalised repetition count (per height and body mass) during the first 10 s (m·kg^−1^)	Normalised rate of force development (per leg length and body mass) at 150 ms (N·m·kg ^−1^·s^−1^)	D	0.15	[−0.34, 0.58]
		ND	0.21	[−0.29, 0.61]
Mean repetition time (s)		D	−0.43	[−0.75, 0.05]
		ND	−0.32	[−0.69, 0.17]
Normalised peak velocity (per height and body mass) during the first five repetitions (m·s^−1^·kg^−1^)		D	0.63 *	[0.21, 0.85]
		ND	0.48 *	[0.01, 0.78]

D: dominant leg; ND: non-dominant leg; correlation significance: * *p* < 0.05.

## Data Availability

The data presented in this study are available from the corresponding author upon reasonable request. Data are not publicly available due to ethical restrictions related to participant confidentiality and the approved study protocol.
